# Exploring immune memory traits in the social immunity of a fungus-growing ant

**DOI:** 10.1098/rspb.2024.1097

**Published:** 2024-12-18

**Authors:** Aryel C. Goes, Pepijn W. Kooij, Ives Haifig, Odair C. Bueno, Andre Rodrigues

**Affiliations:** ^1^ Department of General and Applied Biology, São Paulo State University (UNESP), Rio Claro, Brazil; ^2^ Department of Evolution, Ecology and Organismal Biology, The Ohio State University, Columbus, OH, USA; ^3^ Center for Natural and Human Sciences, Federal University of ABC, São Paulo, Brazil

**Keywords:** immunological memory, hygienic behaviour, pathogens, specificity, attini ants

## Abstract

The immune system is crucial for organisms to defend against pathogens. Likewise, analogous immune features evolved against similar pressures at the superorganism scale. Upregulating hygiene to the same fungus pathogen is one assumption for convergent immune mechanisms in social insects, although more evidence of immune memory features remains to be confirmed. Here, we assess immune memory traits at the colony level in the leaf-cutting ant *Atta sexdens*. We exposed their fungus cultivar to both homologous and heterologous challenges with the entomopathogenic fungi *Metarhizium anisopliae* and *Beauveria bassiana*, as well as the mycoantagonistic fungi *Fusarium oxysporum* and *Trichoderma spirale*. By measuring ants’ behaviours, we evaluated the capacity of *A. sexdens*: (i) to enhance their collective hygiene, (ii) speed their hygiene in further infections, (iii) how long this capacity lasts in the colonies and (iv) the degree of specificity to increase hygienic responses. Fungus grooming behaviour was enhanced mostly against entomopathogenic fungi, with a trend of faster reactions during homologous challenges. In general, the capacity to elicit such upregulated actions lasted for up to 30 days, but no longer than 60 days. Overall, colonies exhibited a degree of immune specificity, enhancing hygiene only in response to homologous exposures but decreasing it when infected secondarily with a different fungus, indicating flexible social immunity of *A. sexdens* after immune challenges.

## 1. Introduction

During the lifetime of an organism, pathogens usually overcome host immunological barriers. Whenever behavioural strategies or external barriers are overcome, innate and adaptive immune responses play decisive roles in defending and reducing damage [1,2]. Inhabiting the same environment throughout a lifetime exposes the host to a high likelihood of infections from the same pool of pathogens. In those cases, maintaining an immune memory would be advantageous [3,4]. The information stored from previous challenges allows the immune system to elicit stronger and faster responses in future encounters with pathogens [3,5]. More specifically, immune memory is a heterogeneous phenomenon of five dimensions reported for both invertebrates and vertebrates [5]: (i) ‘strength’ of reactions upon rechallenge, (ii) ‘duration’ of how long the immune system can still elicit stronger responses (days, weeks, months or years), (iii) the ‘speed’ it takes to increase the reaction upon rechallenge, (iv) ‘specificity’ towards a prior seen target or to a wider spectrum and (v) ‘extinction’ of the immune response (whether the upregulated reaction remains or has disappeared before a re-infection). As emphasized by Pradeu & Du Pasquier [5], these dimensions are gradients that may vary across taxa, meaning that one should not expect similar degrees of strength, specificity, or duration of immune memory within phylum or species. Long thought to be an exclusive feature of the adaptive immune system of vertebrates, immune memory has now been identified in microorganisms, plants and invertebrates [6–8], and as a feature of the innate immune system [9,10]. Thus, it is a phenomenon acknowledged in innate and adaptive immunity with distinct traits, manifesting gradually across its dimensions at the organism level.

Superorganisms, such as ants, termites, some wasps and bees (*sensu stricto* definition [11,12]) possess analogous solutions to those found in the physiological immune system to fight infections, called *social immunity* [13,14]. They police nest entrances to avoid invasion of non-kin individuals, apply chemical and physical defences against pathogens, remove dead or infected individuals, and coordinate their social network to reduce infection [14]. Beyond these mechanisms, the immune memory may also have evolved at the superorganism level to suppress the likelihood of continuous threats [14,15], as stated at the organism level [16–18]. Some ant species enhance their grooming behaviour towards nestmates exposed consecutively to an entomopathogenic fungus [19–21] (i.e. strength), or decrease it when secondarily exposed to a novel pathogen (i.e. specificity) [20]. Such signs of immune memory features also occur for the protection of mutualistic symbionts. The leaf-cutting ant *Atta sexdens* (Linnaeus, 1758) was capable of stronger reactions when exposed three times to the same pathogenic fungus (i.e. strength), eliciting distinct grooming levels towards themselves and to the mutualistic fungus they cultivate [22]. Such evidence highlights the capacity to retain information from past pathogen challenges, suggesting adaptive immune features at the colony level [19,22].

Fungus-growing ants, or ‘attine ants’ (Hymenoptera: Formicidae; subtribe Attina), have significant potential to exhibit traits of collective immune memory. They share the nest space with a symbiotic mutualistic fungus which is seen as the external digestive system of the colony [23,24], since it breaks down the plant material the ants cannot digest themselves and rewards the ants with nutrients. Because the ants cultivate fungal mutualists as a food source [25,26], foraging for fresh or dry plant material to manure the fungus introduces a myriad of potentially antagonistic microbes into their nests [26–31], requiring additional hygienical strategies to protect and clean both themselves and the fungal cultivar [31–33]. Given the likelihood of the superorganism (i.e. kin individuals and mutualistic symbionts) facing threats across its lifetime [34], one might expect immune memory traits at the group level to evolve in response to pressures from various pathogens in fungus-growing ants. This would promote more specific, stronger and efficient responses in secondary challenges [14,33]. While some leaf-cutting ants, such as *Acromyrmex echinatior* (Forel, 1899) [19] and *A. sexdens* [22], show intensified hygienic responses to successive pathogen exposures, other aspects of immune memory still need confirmation. These include species-specific increases in hygiene, the speed of response in secondary challenges and the duration of this enhanced capacity (short term or long term).

Building on previous social immunity studies with attine ants [19,22], we investigated which immune memory traits the leaf-cutting ant *A. sexdens* possesses at the colony level. We explored four aspects of Pradeu & Du Pasquier’s definition of immune memory [5]: (i) is hygiene higher in secondary immune challenges (strength)? (ii) Does the increase in hygienic behaviour occur more rapidly in secondary immune challenges (speed)? (iii) How long does the enhanced response capacity persist (duration)?, and (iv) does enhanced hygiene only occur to a previously exposed pathogen (specificity)? To assess these traits, we exposed fungus gardens of *A. sexdens* to both homologous and heterologous challenges and measured the ant hygienic responses. We used two entomopathogens, *Beauveria bassiana* (Bals.-Criv.) Vuill. (1912) and *Metarhizium anisopliae* (Metschn.) Sorokin (1883)*,* as well as two potential antagonists to the fungus cultivar commonly found in attine ant colonies, i.e. *Fusarium oxysporum* Schltdl. (1824) and *Trichoderma spirale* Bissett (1992), and measured group hygiene over time.

## 2. Material and methods

### (a) Ant colony maintenance

We collected in total 80 incipient colonies of the leaf-cutting ant *A. sexdens* at the Itirapina Ecological Station, São Paulo, Brazil (-22.225662-47.840134), in the summer of 2021 and reared them at the Centro de Estudos de Insetos Sociais (CEIS, UNESP, Rio Claro, State of São Paulo, Brazil). The fungus garden containing the queen, workers and offspring was kept in an acrylic glass container (16.5 cm length × 11 cm width × 7 cm height) with 1 cm of plaster on the bottom to maintain humidity. The container was connected to two smaller-sized containers (10 cm in diameter and 5 cm height, each) for the foraging area and the dump chamber. Each colony was allowed to settle and grow for 4 months before the start of the experiments. The colonies were maintained at approximately 24°C with a daylight regime (12 h:12 h light-dark cycle). Fresh leaves of *Mangifera indica* L. (1753) and *Syzygium jambos* (L.) Alston (1931) were offered alternately every other day.

### (b) Fungal cultures and conidial suspension

Aiming to investigate whether *A. sexdens* colonies exhibit immunological memory traits, one strain of *B. bassiana* LESF 477 and one of *M. anisopliae* LESF 206 were used to represent a threat to the ants, i.e. entomopathogens [35,36]. One strain each of *F. oxysporum* LESF 333 and *T. spirale* LESF 117 was chosen due to their capacity to harm the fungus cultivar, as they are considered mycoantagonists in this context [29,37]. These four strains elicited sanitary care from the ants in a previous study [22]. All fungal cultures used in this study were deposited in the collection of the Laboratory of Fungal Ecology and Systematics (LESF, UNESP, Rio Claro, State of São Paulo, Brazil). The fungi were cultivated and maintained in Petri dishes containing potato dextrose agar medium (PDA: 20 g l^–1^ of agar, 4 g l^–1^ of potato extract and 20 g l^–1^ of dextrose; Neogen^®^ Culture Media, Lansing, MI, USA), supplemented with 150 µg ml^–1^ of chloramphenicol (Sigma-Aldrich, St. Louis, MO, USA). Plates were incubated at 25°C in the dark for 10 days. Prior to the experiments, the fungi were transferred to a new Petri dish with PDA and incubated at 25°C.

We prepared the conidia suspensions by collecting hyphae and conidia from 7 day old cultures [38]. We suspended the material in 0.05% Triton X (Sigma-Aldrich, St. Louis, MO, USA) diluted in water in sterile 10 ml plastic tubes. To separate the mycelium from the conidia, we vortexed the material for 1 min and then filtered the suspensions using a 0.5 µm sterile filter membrane (Millipore Sigma, Burlington, MA, USA) for *B. bassiana,* and a 0.40 µm sterile cell strainer (Sigma-Aldrich, St. Louis, MO, USA) for the other fungal species. The concentration of conidial suspension was measured with a haemocytometer (HBG^®^, Gurugram, Haryana, India) and diluted to approximately 10^6^ conidia ml^−1^. For all experiments, we used a sterile 5 ml hand sprayer to apply 0.5 ml of the conidial suspensions or 0.5 ml of 0.05% Triton X (sham solution) solely to the fungus garden. Before reusing the sprayers, we immersed both the nozzle and the tube in 96% ethanol for 2 days and gently washed with distilled water. We checked the conidia viability on Petri dishes with PDA, incubated at 25°C for 48 h. After incubation, we found over 97%–98% viability in all cases.

### (c) Homologous and heterologous challenges

**Figure 1.**
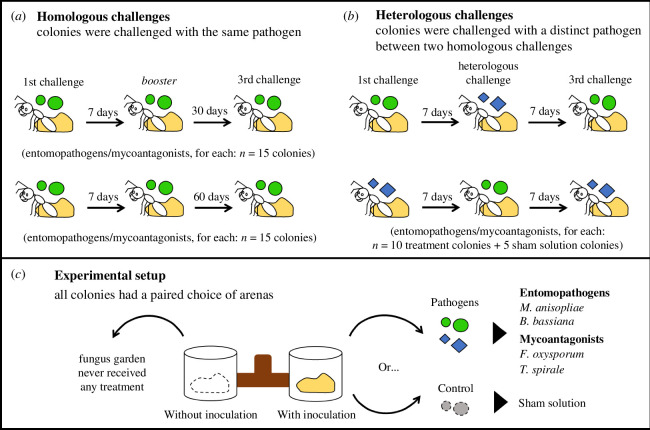
Experimental design of the homologous and heterologous challenges. The bioassays were divided into two major groups according to pathogen lifestyle: entomopathogens (*Beauveria bassiana* and *Metarhizium anisopliae*) and mycoantagonists (*Fusarium oxysporum* and *Trichoderma spirale*). (*a*) To evaluate the increase of cleaning responses to a given pathogen (strength) and how fast they are applied (speed), homologous challenges were introduced to colonies containing a first exposure and a 7 day interval for a booster challenge. After that, a third delayed challenge was applied to check how long the memory lasts (duration). The delayed challenge divided the homologous bioassays into two sets of colonies to evaluate how long the collective immune memory traits last in colonies: one set received the third challenge after a 30 day interval after the booster exposure and the second set of colonies after a 60 day interval. (*b*) To pursue the degree of specificity in collective immune memory, heterologous challenges were applied in colonies within 7 day intervals. (*c*) During assay days, each colony had attached the experimental set-up that led to a paired choice of arenas: one arena received a fungus garden that had no inoculation, and the other one received a pathogen inoculation (on treatment colony) or a control inoculation (on sham solution colonies).

Based on the fungal lifestyle, we divided homologous and heterologous assays into two major groups: entomopathogens (*B. bassiana* and *M. anisopliae*) and mycoantagonists (*F. oxysporum* and *T. spirale*). To check whether *A. sexdens* colonies respond faster and stronger towards secondary exposures, we inoculated the fungus garden with the same pathogen in a first challenge and, after 7 days, we inoculated a ‘booster’ challenge (homologous challenge; figure 1*a*). This ‘booster’ challenge was applied only once. The 7 day interval was chosen to allow the colonies to experience and recover from later infection caused by entomopathogens [35]. We used five colonies for each pathogen and five colonies to receive the sham solution: five colonies for *B. bassiana +* five colonies for *M. anisopliae* + five colonies for the sham solution (as the entomopathogen group, *n* = 15); five colonies for *T. spirale +* five colonies for *F. oxysporum* + five colonies for the sham solution (as the mycoantagonist group, *n* = 15). Since we wanted to check whether the enhancement in hygiene is long-lasting (duration dimension), we repeated the same homologous experiment with two additional sets of colonies: one set received a third homologous challenge 30 days after the ‘booster’ challenge (entomopathogen/mycoantagonist, for each = 15 colonies), while the other set received the third challenge after 60 days (entomopathogen/mycoantagonist, for each = 15 colonies). We chose these time lags based on the age polyethism of major and medium workers of *A. sexdens,* since they are both assigned to gardening and foraging activities after 9 and 4 weeks, respectively [39]. Because the switch of tasks by age affects the distribution of workers [39], social immune responses may be affected by the availability of experienced and naive workers to respond to future infections.

To evaluate whether the ants enhance their hygiene specifically to previously met pathogens, we subjected colonies to a heterologous challenge with 7 day intervals (heterologous challenge; figure 1*b*). For each major group, five colonies received a first exposure of pathogen A, followed by a heterologous challenge with pathogen B and a reintroduction of pathogen A in a third challenge. Five other colonies received a contrasting order: pathogen B, pathogen A and pathogen B (entomopathogens/mycoantagonists, for each: *n* = 10 treatment colonies + 5 sham solution colonies; figure 1*b*). We chose this challenging sequence to: (i) reveal the degree of specificity in *A. sexdens* to enhance immune responses to single or multiple pathogens, and (ii) to address whether the enhanced hygiene happens regardless of the fungal conidia the ants are being exposed to. Simplified, stronger and faster responses would only be elicited towards a previously seen pathogen, and not be affected by a challenge of a different pathogen, denoting a level of specificity in immune memory [3,17].

### (d) Experimental set-up and inoculation procedure

On the challenge days, we disconnected the feeding arena of each colony and replaced it with a polyvinyl chloride-tee (2.5 cm in diameter), which led to a bifurcation of two isolated plastic pots (10 cm in diameter and 8 cm height, each) with 1 cm layer of plaster on the bottom. We placed a piece of fungus garden of similar size (2.5–3 cm^3^) from the main cultivar matrix in each chamber 1 day before the challenge. Only one chamber received the treatment (conidia or sham solution) and the other one was a negative control, i.e. without any sort of inoculation (figure 1*c*). The fungus garden without inoculation was used to check whether manipulation would bias cleaning actions. Since we followed the initial defensive responses from zero to a 1 h period and aimed to see the recruitment progress, we removed all the ants, brood and queen from the fungus fragments. With soft forceps, we removed all the ants and brood from the piece while avoiding disrupting the whole garden structure. We left minima workers (i.e. gardening caste) to maintain the cultivar’s health overnight since it cannot be left unattended for hours (AC Goes, 2022, personal observation). On the next day, before the start of the experiment, we carefully removed reminiscent workers from both garden pieces. For each colony, these procedures did not exceed 10 min to minimize fungus desiccation.

In nature, plant material brought into the nest to feed the fungus cultivar can introduce and contaminate the colony with fungal spores from the environment [27–30]. Our challenge procedures aimed to simulate a natural threat that *A. sexdens* may find in the colony, after the detection of contaminants in the fungus garden [31], and measure workers’ differential reactions towards an entomopathogen or a mycoantagonist fungi in the nest. Hence, in all assays, only the fungus garden fragment received the treatment solutions (conidial suspension or sham solution), while the ants were never directly infected. We applied the treatment solution in one of the two fungus pieces based on a previous raffle. We distinguished the challenge arena by a simple ink mark on the lid/side of the chamber. After that, we immediately connected the set-up to the respective colony and started the video recording of both arenas. All treatments were performed at the same time of day, i.e. between 07.00 and 11.00 UTC −3. After 1 h, we disconnected the T-junction and returned all the reminiscent workers to their respective colonies. These manipulations were performed carefully to avoid losing the experienced workers or accidentally killing them. We never reused the fungus fragment for the next exposure but rather discarded it to avoid bias.

At the end of each challenge, the detached experimental chambers were soaked for 2 days in the neutral detergent Extran 5% (Merck KGaA, Darmstadt, Germany), washed and air-dried for further use in the next challenges. We always replaced the plaster and surface sterilized the two arenas with UV light for 15 min. We cleaned the experimental chambers to remove any chemical and trace odours left by ants from the previous challenge. To avoid bias in ants’ choices and actions based on spatial information retained by workers from the previous challenge, we switched the location of fragments with and without inoculation in further exposures.

### (e) Video recording and scoring behaviours

The video recording started immediately after the attachment of the T-junction to the main colony. We followed the progression of cleaning behaviours toward the challenged and non-challenged pieces of the fungus cultivar, throughout 1 h. We recorded fully the first 10 min (i.e. lag time), which is the average time required for workers from the main arena to explore the experimental set-up, detect and recognize the treatment offered. Then, we continued recording the arenas at 15, 30, 45 and 60 min, with video recordings of 15–20 s long. Evaluating the dynamic of defensive responses through these intervals is important to understand whether the colonies were faster in secondary exposures. We used two Sony HDR – CX150/B (9.0 megapixels) video cameras and allocated them to one arena each from the experimental set-up. Thus, colonies were recorded individually. We managed to record the fungus garden fragment and the whole area of the plastic pots. We always randomized the sequence of recordings, alternating between colonies that received the pathogen or the sham solution.

A single examiner (A.C.G.) analysed all the video recordings counting the number of: (i) defensive behaviours displayed by workers interacting with the fungus fragment, (ii) the total number of ants on the fragment and (iii) the total number of workers in the arena. We used a hand counter (VMC-4 model, Vonder, Curitiba, PR, Brazil) to facilitate the counting. We chose the instantaneous scan sampling for assessments. In short, during the video, the examiner scored the behaviour presented by each individual at the time it was observed [40]. The same individual was never observed twice. To avoid and reduce bias during the video assessment, another person (P.W.K.) renamed the video files for a blind assignment [41]. We scored the frequency of two hygienic behaviours from *A. sexdens’* social immunity (i.e. fungus grooming and self-grooming; we did not notice allo-grooming and metapleural gland grooming in all bioassays) as described previously in Nilssøn-Moller *et al*. [32] and Goes *et al*. [22], and a singular defensive behaviour seen in our study (fungus rescue):

- *Fungus grooming*: when an ant was immobile at a fixed point of the fungus fragment. The antennae remained motionless and parallel to the fungus, with the ends touching the tips of the mandibles. The ant opened its mandibles and did retracting movements with the head, pulling off a tiny portion of the fragment, or the glossa licked the fungus surface.
- *Fungus rescue*: when an ant uses its mandibles to either cut or detach a large piece of the fungus fragment, pulling it off and carrying it out from the arena. We also scored this defensive behaviour whenever an ant was found carrying the fungus piece inside the arena. The ant usually returned the fragment to the main arena, putting it back in the fungus garden matrix (AC Goes, 2022, personal observation). Therefore, we considered this behaviour different from the previously described ‘fungus weeding’ since the fragments were not discarded in the dump.
- *Self-grooming*: when a single ant remained at a portion of the fungus fragment and brushed the antennae on the front legs; or when the ant cleaned the antennae and the legs by passing them through the mouthparts, removing particles with their glossa, several times.

### (f) Statistical analyses

Most of our data were based on ‘fungus grooming’ responses, whereas ‘self-grooming’ was a small representative (electronic supplementary material, figure S1). Thus, we pooled both cleaning behaviours data into a single category called ‘total number of cleaning responses’, assuming that this does not bias the conclusions of our study. Besides, we considered each *A. sexdens* colony as a single superorganism that would experience and respond in a group-level dimension, reinforcing our interest in analysing all the social immunity elements together. Nevertheless, because specific behaviours are expected to be applied to specific types of threats (e.g. more self-grooming against an entomopathogenic fungus), we also ran separate analyses for each behaviour category (see electronic supplementary material for these analyses). Lastly, we considered the fungus rescue behaviour as a defensive rather than a cleaning strategy. We observed this towards fragments that did not receive inoculation and in a low frequency (electronic supplementary material, figure S2). Ants recovered the unattended piece of the cultivar and brought it back to the main fungus matrix. We did not consider this as a cleaning strategy for analysis because ants also took infected pieces back to the main colony, introducing a threat to them.

The pooled data did not have a normal distribution. Thus, to assess whether the amount of cleaning varied within challenges, we applied the generalized linear mixed-effects model analysis (GLMM [42]; glmmTMB package [43]). To select the best model, we applied the Akaike information criterion (AIC) to compare models with distinct factors and the following family distributions: Poisson, Conway–Maxwell Poisson, Negative Binomial 1 and Negative Binomial 2, with the default link function. We also checked the good fitting of the model using residual diagnostics for hierarchical regression (DHARMa package [44]), accounting for overdispersion and zero-inflation (electronic supplementary material, tables S1 and S2, for chosen models).

For modelling homologous and heterologous assays, the number of cleaning responses was the response variable. Time intervals (5, 10, 15, 30, 45, and 60 min), the arena where the responses came from (with or without inoculation; figure 1*c*), and the interaction between treatment and challenge were used as fixed effects. The ant colony nested within challenge, and nested within time intervals were used as the random effects because they represent multiple measures without independence [45]. The best-fitted distribution was Negative Binomial 1 for all fitted models (electronic supplementary material, table S1). Whenever we found a significant fixed effect, we performed *post hoc* tests to determine whether prior or later challenges had a significant increase/decrease in comparison to each other, using Tukey’s test (emmeans package [46]).

To investigate whether hygiene was faster through homologous challenges (figure 1*a*), we considered the number of ants visiting the arena as the response variable. We argue that the trend of more cleaning behaviours in less time through challenges might be dependent on the number of ants visiting the arenas. Thus, we ran models considering the interaction between treatment and challenge, the time intervals and the arenas as fixed effects; the colony nested within challenge and time were the random factors. The best-fitted distribution was Negative Binomial 1 for all the models. Whenever necessary, we accounted for zero-inflation and overdispersion in the model with ‘zi’ and ‘dispformula’ from the package glmmTMB [43] (see electronic supplementary material, table S2, for chosen models). We also checked whether cleaning responses varied due to the effect of time in the prior analyses with the cleaning as the variable response.

We applied likelihood ratio tests with the ‘anova’ function to compare a model carrying the interaction of a fixed effect against a null model, i.e. without the aimed interaction. Then, we could determine whether the interaction between treatment and challenge significantly explained the variation of the response variable. Statistics were carried out in R 4.0.3 [47], and scripts used are available at Figshare (https://doi.org/10.6084/m9.figshare.23631981). Plots and graphs were created with the functions ‘ggplot2’ [48] and ‘dplyr’ package [49].

## 3. Results

*A. sexdens* colonies exhibited more cleaning behaviours in the arenas with inoculation (electronic supplementary material, figure S3*a*,*b*), whereas fungus rescue was observed often in the arenas without inoculation (electronic supplementary material, figure S2*a*,*b*); ants tended to bring back the fungus fragment from arenas without inoculation to the main colony, ‘planting’ it back in the main fungus garden. Fungus grooming and self-grooming were elicited when challenged with entomopathogens, mycoantagonists and the sham solution (electronic supplementary material, figure S1). There was a significant interaction between the treatments and the behaviour applied that explains our variable response through assays (GLMM: *χ*² = 29.447, d.f. = 1, *p* = 0.001; comparison to the null model: *χ*² = 915.5, d.f. = 3, *p *< 0.001). Therefore, the amount of fungus grooming and self-grooming varied between entomopathogens and mycoantagonists, with a trend of more self- and fungus grooming against entomopathogens (electronic supplementary material, figure S1*a*,*b*). Fungus grooming was substantially applied by ants and accounted for most of the hygiene observed (electronic supplementary material, figure S1*a*), whereas self-grooming was less applied (electronic supplementary material, figure S1*b*). Overall, colonies increased fungus and self-grooming when challenged secondarily with the same entomopathogenic and mycoantagonistic fungi, even after a 30 day interval; decreases in fungus grooming in heterologous challenges with both types of fungi were seen (electronic supplementary material, table S3).

### (a) Enhanced hygiene towards homologous challenges

**Figure 2.**
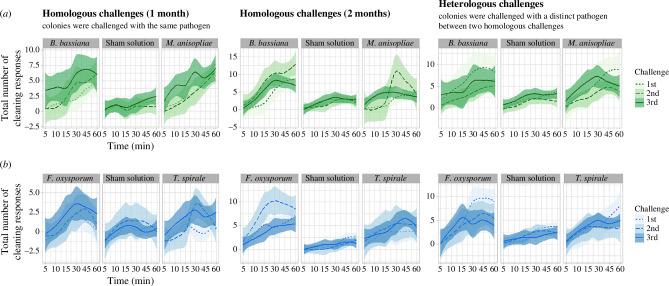
Total number of cleaning responses over time and challenge: (*a*) entomopathogenic fungi (*Beauveria bassiana* and *Metarhizium anisopliae*)*,* (*b*) mycoantagonistic fungi (*Trichoderma spirale* and *Fusarium oxysporum*) and the sham solution. Distinct lines represent distinct challenges, while the colour shades represent confidence intervals. Sanitization was similar between the first and booster challenges of the same entomopathogen, except for the booster challenge of *B. bassiana*. Colonies triggered higher hygiene in the third challenge after 30 days, but not after 60 days. Sanitization showed a non-significant trend to increase in secondary challenges to the same mycoantagonistic fungi even after 30 days, although a significant decrease was seen against a third challenge of *F. oxysporum* after 60 days. There was a trend to decrease hygiene against the heterologous challenge with entomopathogens in comparison to the first and third one, whereas there is no suggestion of this with the mycoantagonists. As depicted: ‘1st’ = first challenge, ‘2nd’ = second challenge (booster for homologous assays; heterologous challenge for heterologous assays) and ‘3rd’ = third challenge (memory challenge for homologous assays).

**Figure 3.**
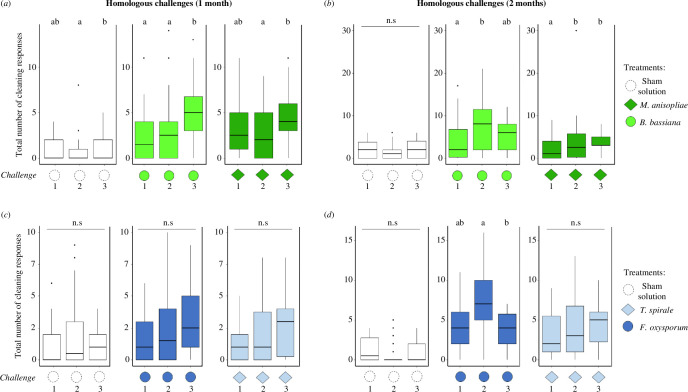
Number of cleaning responses in the homologous assays, for each challenge, against entomopathogens (*Beauveria bassiana* and *Metarhizium anisopliae*)*,* mycoantagonists (*Trichoderma spirale* and *Fusarium oxysporum*) or the sham solution. Sanitization intensity was similar between the first and second challenges (booster) of the same entomopathogen in the 1 month assay (*a*) but showed a significant increase in the 2 month assay (*b*). Colonies also showed higher hygiene in a third challenge after 30 days (*a*). The same occurred after 60 days, but only towards *M. anisopliae* (*b*). There was a non-significant trend of enhancement in secondary challenges to the same mycoantagonist even after 30 days (*c*), although a significant decrease was seen against the third challenge of *F. oxysporum* after 60 days (*d*). The sham solution usually did not elicit substantial cleaning. Letters indicate significant differences (*post hoc* at *α* = 0.05). ‘n.s’ stands for no significant difference.

The number of cleaning behaviours varied throughout time intervals in the 1 month assays of entomopathogens (GLMM: *χ*² = 136.1531, d.f. = 5, *p* < 0.001; figure 2*a*), with a higher frequency in the arenas that received treatments than in those that did not (*z*-value = 9.390, *p* < 0.001; electronic supplementary material, figure S3*a*). The interaction of treatments and challenges was not significant in this assay (GLMM: *χ*² = 7.3375, d.f. = 4, *p* = 0.119), reinforced by the *post hoc* comparisons that show no significant enhancement of hygiene in the booster challenge 7 days later with *M. anisopliae* (Tukey *post hoc*, challenge 1 versus challenge 2: *p* = 0.644; figure 3*a*)*, B. bassiana* (Tukey *post hoc*, challenge 1 versus challenge 2: *p* = 0.646; figure 3*a*) and the sham solution (Tukey *post hoc*, challenge 1 versus challenge 2: *p* = 0.087; figure 3*a*).

In the 2 month homologous assay with entomopathogens, the amount of hygiene was affected throughout time intervals (GLMM: *χ*² = 178.131, d.f. = 5, *p* < 0.001; figure 2*a* and S4), being more frequent in the arenas with inoculation (*z*-value = 13.131, *p <* 0.001; electronic supplementary material, figure S3*a*). Hygiene was affected by the interaction of treatments and challenges (GLMM: *χ*² = 10.594, d.f. = 4, *p =* 0.0315), showing a significant enhancement in the booster exposure of *B. bassiana* (Tukey *post hoc*, challenge 1 versus challenge 2: *p =* 0.041; figure 3*b*) and *M. anisopliae* (Tukey *post hoc*, challenge 1 versus challenge 2: *p =* 0.031; figure 3*b*). There was no difference comparing first/booster challenges for the sham solution (Tukey *post hoc*, *p =* 0.972; figure 3*b*).

The total hygiene varied throughout the time intervals considering all treatments in the 1 month homologous assay with mycoantagonists (GLMM: *χ*² = 68.9641, d.f. = 5, *p* < 0.001; figure 2*b*). Such number of behaviours occurred significantly only in the arenas with inoculation (*z*-value = 13.262, *p* < 0.001; electronic supplementary material, figure S3*b*), although no impact by treatment and challenge was observed (GLMM: *χ*² = 1.0147, d.f. = 4, *p* = 0.907). In addition, there was no indication of enhancement between first/booster challenges towards all treatments (Tukey *post hoc*, challenge 1 versus challenge 2: *p >* 0.05 for all; figure 3*c*).

Sanitization significantly varied throughout time intervals in the 2 month assay with mycoantagonists (GLMM: *χ*² = 92.428, d.f. = 5, *p* < 0.001; figure 2*b* and S5), specifically in the arenas that received treatments (*z*-value = 14.172, *p* < 0.001; electronic supplementary material, figure S3*b*). Sanitization was affected by the interaction of treatments and challenges (GLMM: *χ*² = 13.741, d.f. = 4, *p =* 0.008), although colonies did not elicit higher hygiene in booster challenges of all treatments (Tukey *post hoc*, challenge 1 versus challenge 2: *p >* 0.05 for all comparisons; figure 3*d*).

### (b) Trend for faster responses in homologous challenges

Against entomopathogens, the number of ants visiting the arenas significantly varied through time (GLMM: 1 month: *χ*² = 167.5670, d.f. = 5, *p* < 0.001; electronic supplementary material, figure S6*a*; 2 month: *χ*² = 203.6680, d.f. = 5, *p* < 0.001; electronic supplementary material, figure S6*a*). The number of ants flowing in the arenas with and without inoculation was similar in the 1 month assay (1 month: 0.650, *p =* 0.516; electronic supplementary material, figures S6*a* and S7*a*), whereas a higher number in the arena with inoculation was observed in the 2 month assay (2 month: *z*-value = 2.231, *p =* 0.025; electronic supplementary material, figure S6*a*). Cleaning actions and recruited ants seemed higher in the first minutes against a memory challenge 30 days later (figure 2*a* and electronic supplementary material, figure S6a), i.e. in less time in comparison to previous challenges. Although the number of ants tended to increase through challenges (electronic supplementary material, figure S6*a*), the number of cleaning reactions remained similar, with a drop in the memory challenge 60 days later (figure 2*a*).

Sanitizations against mycoantagonists were also impacted by time (GLMM: 1 month: *χ*² = 148.5927, d.f. = 5, *p* < 0.001, electronic supplementary material, figure S6*b*; 2 month: *χ*² = 99.2036, d.f. = 5, *p* < 0.001; electronic supplementary material, figure S6*b*), with the total number of ants being higher in the arena with inoculation only in the 1 month assay (1 month, *z*-value = 4.068, *p* < 0.001; 2 months, *z*-value = −0.395, *p* = 0.692; electronic supplementary material, figures S6*b* and S7*b*). Overall, the number of ants in the treatment arenas maintained similar over challenges and time intervals (electronic supplementary material, figure S6*b*), except for more ants cleaning in less time at the third challenge of *F. oxysporum* and *T. spirale,* 30 days later (figure 2*b*). In the 2month assay, a singular peak of hygiene occurred between 15 and 45 min in the second challenge of *F. oxysporum* (figure 2*b*). The sham solution did not significantly vary (figure 2*b*).

### (c) Increased hygiene responses last up to 30 or 60 days

Colonies were able to trigger upregulated hygiene in a third challenge with the same entomopathogenic fungus after 30 days (Tukey *post hoc*, *B. bassiana* challenge 2 versus challenge 3: *p* = 0.002; *M. anisopliae* challenge 2 versus challenge 3: *p =* 0.018; figure 3*a*). In those colonies that received a third challenge 60 days later, sanitization towards *B. bassiana* remained the same as the previous challenges (Tukey *post hoc*, challenge 1 versus 3: *p =* 0.853, challenge 2 versus 3: *p =* 0.141; figure 3*b*), but was higher against *M. anisopliae* when comparing the first and memory challenge (Tukey *post hoc*, challenge 1 versus 3: *p =* 0.001, challenge 2 versus 3: *p =* 0.595; figure 3*b*). The sham solution had a difference between booster and memory challenges in the 1 month assay (Tukey *post hoc*, *p =* 0.001; figure 3*a*).

Workers did not upregulate hygiene against the same mycoantagonist fungus after 30 days (Tukey *post hoc, p >* 0.05 for all comparisons and treatments; figure 3*c*). In contrast, sanitization significantly dropped when exposed 60 days later to *F. oxysporum* (Tukey *post hoc*, challenge 1 versus 3: *p =* 0.746, challenge 2 versus 3: *p =* 0.024; figure 3*d*), and maintained similar towards *T. spirale* as observed in previous challenges (Tukey *post hoc*, challenge 1 versus 3: *p =* 0.222, challenge 2 versus 3: *p =* 0.676; figure 3*d*). The sham solution did not impact significant differences in hygiene (Tukey *post hoc*, *p >* 0.05 for all comparisons; figure 3).

### (d) Hygienic responses decreased in heterologous challenges

**Figure 4.**
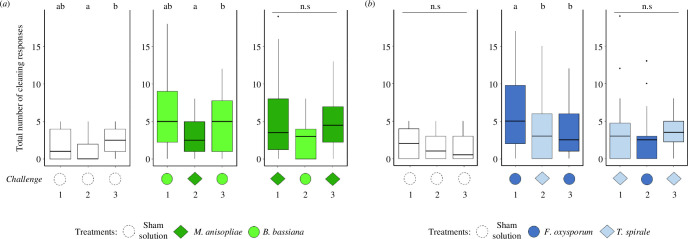
Number of cleaning responses in the heterologous assays, for each challenge, against (*a*) entomopathogenic fungi (*Beauveria bassiana* and *Metarhizium anisopliae*)*,* (*b*) mycoantagonistic fungi (*Trichoderma spirale* and *Fusarium oxysporum*) or the sham solution. Colonies substantially decreased hygiene if they received a heterologous exposure (challenge 2) of *M. anisopliae* between two homologous challenges of *B. bassiana* (challenges 1 and 3). The same occurred with colonies that received a heterologous challenge of *T. spirale* between two homologous challenges of *F. oxysporum.* Letters indicate significant differences (*post hoc* at *α* = 0.05). ‘n.s’ stands for no significant difference.

The number of cleaning behaviours varied throughout time intervals (entomopathogens, GLMM: *χ*² = 121.9868, d.f. = 5, *p* < 0.001; figure 2*a*), with a higher occurrence in arenas that received treatments in comparison to those that did not (entomopathogens: *z*-value = 16.701, *p* < 0.001; electronic supplementary material, figure S3*a*). We found no effect of treatment and challenges interaction on hygiene (entomopathogens, GLMM: *χ*² = 4.4597, d.f. = 4, *p* = 0.347). However, colonies that received a first challenge of *B. bassiana* decreased substantially their hygiene when challenged with *M. anisopliae* 7 days later (Tukey *post hoc*, challenge 1 versus challenge 2: *p* = 0.0004; figure 4*a*). Colonies challenged with *M. anisopliae* followed by a heterologous challenge of *B. bassiana* did not significantly reduce hygiene (Tukey *post hoc*, challenge 1 versus challenge 2: *p* = 0.0915; figure 4*a*). In addition, the number of workers available during the heterologous challenge dropped or remained similar to the first exposure (electronic supplementary material, figure S6*a*). We did not observe elicited hygiene in colonies challenged again with the original pathogen seen 14 days earlier (Tukey *post hoc*, *M. anisopliae* challenge 1 versus challenge 3: *p* = 0.1546; *B. bassiana* challenge 1 versus challenge 3: *p =* 0.055; figure 4*a*). The sham solution triggered more hygiene in the third challenge (Tukey *post hoc*, challenge 2 versus challenge 3: *p* = 0.0155; figure 4*a*).

Hygiene also varied through time in the heterologous assay with mycoantagonists (GLMM: *χ*² = 161.3087, d.f. = 5, *p < *0.001; figure 2*b*), occurring significantly in the arenas that received inoculation (*z*-value = 16.957, *p <* 0.001; electronic supplementary material, figure S3*b*). The interaction between treatments and challenges was not significant in this assay (mycoantagonists, GLMM: *χ*² = 5.9277, d.f. = 4, *p =* 0.204). Colonies that received a first challenge with *F. oxysporum* reduced their hygiene when later challenged with *T. spirale* (Tukey *post hoc*, *F. oxysporum* challenge 1 versus challenge 2: *p =* 0.0387; figure 4*b*), and even when received again *Fusarium* in a third challenge (Tukey *post hoc*, challenge 1 versus challenge 3: *p =* 0.0220; figure 4*b*). Cleaning was not impacted in colonies that had a heterologous challenge of *F. oxysporum,* nor when received again *T. spirale* in a third challenge (Tukey *post hoc*, *T. spirale* challenge 1 versus challenge 2: *p =* 0.2252; challenge 1 versus challenge 3: *p =* 0.2176; figure 4*b*). Lastly, the sham solution also did not significantly impact differences in hygiene (Tukey *post hoc*, sham solution: *p > *0.05 for all comparisons; figure 4*b*). The total number of ants in the arenas remained similar through mycoantagonistic challenges in this assay (electronic supplementary material, figure S6*b*).

## 4. Discussion

### (a) Are hygienic responses higher in secondary challenges?

*A. sexdens* colonies enhanced hygienic behaviours when confronted with the same fungus pathogen in repetitive exposures, consistent with prior studies [19,20,22]. Nevertheless, we only observed this reaction in the third challenge for entomopathogens and the booster of *B. bassiana* at the 2 month assay. Possibly, the first challenge was effective only to elicit enhanced hygiene in the memory challenges. At the same time, the significant increase in the booster of *B. bassiana* could have been a singular and random effect. Alternatively, the 7 day interval between the first and second challenges was too short for ant workers to interact with diseased nestmates [35]. The general lack of enhanced reactivity towards mycoantagonistic fungi could have been due to: (i) the little amount of time that workers had to interact with conidia, i.e. 60 min, (ii) the lack of time to germinate and cause significant harm to be perceived by ants, or iii) *T. spirale* and *F. oxysporum* did not elicit a plastic reaction from the colonies.

Our results mainly show a degree of ‘strength’ for fungus grooming to entomopathogens. For social and immune defences to be adaptive [13,32], one may expect more fungus grooming against fungus garden pathogens, whereas more self- and allo-grooming would be strongly efficient towards ant pathogens, rather than the other way around. However, Yek *et al*. [50] showed that the strategy chosen against fungal pathogens by *Acromyrmex* fungus-growing ants is defined by the context where they found the threat (fungus garden or nestmate) and not the type of pathogen itself. The ants applied fungus grooming when challenging the fungus gardens with conidia of *Metarhizium brunneum* Petch (1935) (an entomopathogen), and allo-grooming when the ants received conidia of *Escovopsis* J.J. Muchovej & Della Lucia 1990 (a mycoantagonist) on their body. The context-dependent response was also seen when the fungus cultivar of *A. sexdens* was challenged with five distinct fungi [22], applying more fungus grooming instead of other cleaning strategies. Thus, fungus grooming in our study can be explained because we inoculated the treatment suspensions only in the fungus garden fragments.

The fungus-growing ant mutualism is a complex system of several organisms tied in an obligate interaction, often considered a superorganism [12,13,23]. Thus, we should consider the fungus and the ants as a single entity under the protection of the social immune system and symbionts [33]. Upregulating fungus grooming against entomopathogens may be important for social immunity based on eco-evolutionary dynamics. Fungus-growing ants rear their offspring surrounded by fungus garden in huge brood chambers [51]. Since entomopathogens are also found in this environment and not only on ant bodies [26,52], any conidia entering the fungus garden is a potential threat to the brood or nursing workers. By applying the challenges to the fungus fragment, we might have triggered an innate behavioural response since the ants may have seen the garden fragment as a part of their brood chamber. These results support context-dependent strategies observed in another fungus-growing ant [50], indicating that the extent of increased sanitization may depend on both the pathogen and the context in which it is encountered.

### (b) Are stronger responses elicited after 30 and 60 days?

The ‘duration’ trait of immune memory is relevant for short- and long-lived organisms with high chances of recurrent infections [3,4]. Considering that *Atta* colonies may live up to 15–25 years in nature [51], they would benefit from long-term memory to elicit plastic responses during their lifetime [53,54]. Our results showed that *A. sexdens* colonies had similar or higher hygiene up to 30 days after the last exposure, and even up to 60 days towards *M. anisopliae* but reduced hygiene towards *F. oxysporum* after such a time interval. Due to the age polyethism of major and medium workers of *A. sexdens,* individuals might change their tasks between the ninth week for maintenance of the fungus garden, and the fourth week for foraging activities [39]. After 60 days of the last challenge (around 9 weeks), experienced workers may have switched their tasks, affecting the availability of workers and hence the strength of the social immune reaction, in contrast to those that received the challenge 30 days later. Although this is the first behavioural study evaluating the duration of plastic hygiene at the colony level, we still lack knowledge on how ants retain long-term information from past infections and whether they are cyclic over time. We hypothesized that stronger actions seen in our study might have occurred due to the sum of individual experience to previous challenges, as in long-term memory for substrate choices in leaf-cutting ants [55,56]. Since workers will perform distinct tasks according to their age [39,57], this could have caused a shift in the number of experienced workers available to respond to further exposures, explaining the observed decline in sanitary responses.

### (c) Are stronger responses faster in secondary challenges?

Faster task performance has been reported in ants for tasks such as thermal brood care [58] and the emigration process [59]. From a social immunity perspective, faster induction of hygiene at the colony level would likely depend on the proportion of ants recruited and available for cleaning tasks, thereby escalating the response more quickly. However, evidence of faster cleaning hygiene following experience remains elusive. Prior studies did not observe a decrease in the time required for detection and reaction against *Metarhizium* Sorokin 1879 challenges in the clonal ant *Platythyrea punctata* (Smith, 1858) [21], nor did they find faster removal of fungus-contaminated waste in *Myrmica rubra* (Linnaeus, 1758) [60]. Instead, these ants appeared to spend more time removing contaminants and increasing the number of collective actions. In contrast, *A. sexdens* demonstrated some degree of the ‘speed’ trait, as cleaning increased after half an hour under homologous challenges against all fungi in the 30 day challenge, with a higher number of ants in the arenas in less time. Further studies could build upon our results by evaluating how long it takes for *A. sexdens* or other social insects to detect conidia and start the recruiting of nestmates to escalate hygiene in secondary challenges [61], and whether the ‘speed’ trait observed here is based on group-level experience due to consecutive encounters of the same pathogen.

### (d) Do increased responses mean specificity?

‘Specificity’ is another remarkable trait of immune memory. The traditional perspective is that immune memory is specific [3,5]. Yet, stronger responses may or may not come with specificity [16,17]. A degree of specificity will be ascertained if faster and enhanced responses only occur to a previously met pathogen, as stated for immune priming [3,62]. *A. sexdens* did not express stronger hygiene when confronted with a second distinct pathogen but rather decreased it, regardless of the fungus they were exposed to. This highlights their capacity to: (i) discriminate between two distinct infection events, decreasing actions to a novel pathogen and (ii) that enhanced responses were not induced by the challenge itself but by a prior infection to a singular fungus [22]. Collateral damage [14] and costly allocation of resources [54] are examples of social immunity trade-offs for a colony’s well-being [14]. Therefore, avoiding triggering intense hygiene to an unknown secondary infection might prevent unnecessary loss of fungus garden by excessive fungus weeding by these species [31] or antimicrobials. Lastly, the decrease in social immune responses in heterologous challenges may avoid superinfection of workers that were exposed before, an effect observed in the invasive ant *Lasius neglectus* Van Loon, Boomsma & Andrásfalvy 1990 [20].

Still, the degree of specificity in *A. sexdens* remains uncertain. We can speculate that this species might not exhibit a high level of specificity [22], as ants did not show stronger hygiene responses when re-exposed to a previously encountered pathogen. This suggests that a novel infection might not affect the optimal responses to experienced pathogens. Further studies with different fungal species and strains will be needed to assess how specific *A. sexdens’* social immunity is and how it allocates defensive resources in future infections.

## 5. Conclusions

Organisms and superorganisms (such as ants) must confront pathogens during their life. Thus, besides molecular and cellular immune responses, superorganisms have evolved collective defences to control infections. By showing similarities to the physiological immune system, social immunity became a research focus for potential evolutionary roots of the immune system. Our study is the first to evaluate four traits that compose immune memory at the colony level. Stronger and seemingly faster responses up to 30 days after the last challenge to the same pathogen, and specificity for such plasticity, are novel features covered and reported for the leaf-cutting ant *A. sexdens*. Since these features were mostly supported by fungus grooming against entomopathogens, we understand that we captured a certain degree of these traits under our assay’s conditions. Nevertheless, our results extend knowledge on the flexibility of social immunity mechanisms and the feasibility of experimentally evaluating immune memory traits.

By evaluating these at the colony level, we present the first step towards investigating whether social insects possess ‘social immune memory’. The next phase involves posing questions and designing experiments that assess the impact of these traits on fitness, specifically determining if these and their variations enhance survival and social immune efficiency, indicating their adaptability. We hypothesize that social immune experiences may improve the removal of fungal contaminants, positively affecting group survival or reducing damage during sanitary care. However, based on the responses observed in *A. sexdens* and the lack of information on how these traits influence survival, we cannot yet determine whether the evaluated immune memory traits constitute social immune memory. We can only conclude that this fungus-growing ant exhibits dynamic social immune responses following pathogen challenges.

## Data Availability

The data and codes needed to perform the analysis therein are available in a Figshare repository [63]. All video recordings that resulted in the raw data for this study are publicly available in a Google Drive folder; the URL is in the ‘Read Me’ file in the Figshare repository. Supplementary material is available online [64].
